# Activity of a Long-Acting Echinocandin, Rezafungin, and Comparator Antifungal Agents Tested against Contemporary Invasive Fungal Isolates (SENTRY Program, 2016 to 2018)

**DOI:** 10.1128/AAC.00099-20

**Published:** 2020-03-24

**Authors:** Michael A. Pfaller, Cecilia Carvalhaes, Shawn A. Messer, Paul R. Rhomberg, Mariana Castanheira

**Affiliations:** a JMI Laboratories, North Liberty, Iowa, USA; b University of Iowa, Iowa City, Iowa, USA

**Keywords:** *Aspergillus* spp., CD101, *Candida* spp., antifungal surveillance, antifungal susceptibility testing, echinocandin

## Abstract

We evaluated the activity of rezafungin and comparators, using Clinical and Laboratory Standards Institute (CLSI) broth microdilution methods, against a worldwide collection of 2,205 invasive fungal isolates recovered from 2016 to 2018. *Candida* (*n* = 1,904 isolates; 6 species), Cryptococcus neoformans (*n* = 73), Aspergillus fumigatus (*n* = 183), and Aspergillus flavus (*n* = 45) isolates were tested for their susceptibility (S) to rezafungin as well as the comparators caspofungin, anidulafungin, micafungin, and azoles. Interpretive criteria were applied following CLSI published clinical breakpoints (CBPs) and epidemiological cutoff values (ECVs). Isolates displaying non-wild-type (non-WT) echinocandin MIC values were sequenced for hot spot (HS) mutations. Rezafungin inhibited 99.8% of Candida albicans isolates (MIC_50/90_, 0.03/0.06 μg/ml), 95.7% of Candida glabrata isolates (MIC_50/90_, 0.06/0.12 μg/ml), 97.4% of Candida tropicalis isolates (MIC_50/90_, 0.03/0.06 μg/ml), 100.0% of Candida krusei isolates (MIC_50/90_, 0.03/0.06 μg/ml), and 100.0% of Candida dubliniensis isolates (MIC_50/90_, 0.06/0.12 μg/ml) at ≤0.12 μg/ml. All (329/329 [100.0%]) Candida parapsilosis isolates (MIC_50/90_,1/2 μg/ml) were inhibited by rezafungin at ≤4 μg/ml. Fluconazole resistance was detected among 8.6% of C. glabrata isolates, 12.5% of C. parapsilosis isolates, 3.2% of C. dubliniensis isolates, and 2.6% of C. tropicalis isolates. The activity of rezafungin against these 6 *Candida* spp. was similar to the activity of the other echinocandins. Detection of the HS mutation was performed by sequencing echinocandin-resistant or non-WT *Candida* isolates. Good activity against C. neoformans was observed for fluconazole and the other azoles, whereas the echinocandins, including rezafungin, displayed limited activity. Rezafungin displayed activity similar to that of the other echinocandins against A. fumigatus and A. flavus. These *in vitro* data contribute to accumulating research demonstrating the potential of rezafungin for preventing and treating invasive fungal infections.

## Abstract (precis)

We evaluated the activity of rezafungin and comparators, using Clinical and Laboratory Standards Institute (CLSI) broth microdilution methods, against a worldwide collection of 2,205 invasive fungal isolates recovered from 2016 to 2018. *Candida* (*n* = 1,904 isolates; 6 species), Cryptococcus neoformans (*n* = 73), Aspergillus fumigatus (*n* = 183), and Aspergillus flavus (*n* = 45) isolates were tested for their susceptibility (S) to rezafungin as well as the comparators caspofungin, anidulafungin, micafungin, and azoles.

## INTRODUCTION

Among the available systemically active antifungal agents, the echinocandins, including caspofungin, anidulafungin, and micafungin, and the azoles, such as fluconazole, voriconazole, isavuconazole, and posaconazole, are all employed empirically as directed therapy and for prophylaxis in patients with suspected or documented invasive fungal infection (1–7). Whereas fluconazole remains the most frequently employed antifungal globally, the use of the echinocandin class has steadily increased in academic and community hospital settings (2, 4, 7–11).

The documented potency, spectrum, and safety of the echinocandins have led many experts in infectious diseases to consider echinocandins to be initial therapy for treating candidemia (5, 7, 9, 12). A meta-analysis of randomized clinical trials comparing treatment for candidemia and invasive candidiasis (IC) showed that initial therapy with an echinocandin was a significant predictor of survival (13). Once clinically stable, de-escalation to an oral azole (usually fluconazole) is suggested for all patients (1, 5, 7, 12, 14).

Echinocandins have some important limitations, despite their proven safety and efficacy (9, 15). Most notably, the daily parenteral dosing requirement complicates administration postdischarge in patients requiring extended therapy. Indeed, much of the growth in outpatient antifungal expenditure, as documented in a recent survey of antifungal use in U.S. hospitals, was for echinocandins. This survey suggests that outpatient antifungal use may be increasing (7). Although step-down therapy from an echinocandin to fluconazole may partially address the outpatient antifungal expenditure, it is complicated by the increasing rates of resistance to fluconazole among common species of *Candida* (1, 7, 9, 14, 16). Other potential drawbacks of the available echinocandins for clinical application are the use of a fixed dose irrespective of body size or species susceptibility (S) and emerging resistance mediated by mutations in the *fks* genes (15, 17). It has been suggested that underdosing of echinocandins, coupled with poor penetration to certain body sites, may partially account for the emerging echinocandin resistance (15, 18, 19). An echinocandin that could be safely administered at higher doses to ensure optimal pharmacokinetic (PK) and pharmacodynamic (PD) features and target attainment may facilitate outpatient therapy, reduce the hospital stay, and possibly delay or prevent the development of echinocandin resistance, thus becoming an important step toward improving the ability to effectively manage candidemia and IC (15, 20).

Rezafungin (Cidara Therapeutics, Inc.) is a novel echinocandin that exhibits a prolonged half-life and that displays chemical stability in plasma, in aqueous solution, and at an elevated temperature (15, 21–27). The *in vitro* activity of rezafungin against *Candida* spp. has been shown to be comparable to that of other clinically available echinocandins (2, 28–36). Rezafungin is being developed for the treatment of candidemia and other forms of IC by once-weekly intravenous administration (27). A phase 3, randomized, double-blind, multicenter clinical trial of the efficacy and safety of rezafungin for injection compared with those of intravenous caspofungin followed by oral fluconazole step-down in the treatment of subjects with candidemia and/or IC (ClinicalTrials.gov registration number NCT03667690; ReSTORE) is under way.

In the present study, we examined the *in vitro* activity of rezafungin compared with that of the other systemically active antifungal agents by testing a global collection of 2,205 clinical isolates of yeasts (*Candida* and *Cryptococcus* spp.) and molds (*Aspergillus* spp.) obtained during the 2016 to 2018 SENTRY Surveillance Program. All isolates were submitted to broth microdilution (BMD) susceptibility testing following Clinical and Laboratory Standards Institute (CLSI) methods (37, 38).

(Some results have been presented, in part, for the individual years included in the study period, at the following scientific conferences: ASM Microbe 2018 [34], IDWeek 2018 [35], and IDWeek 2019 [36].)

## RESULTS

### Geographic distribution of *Candida* species.

Among the 1,904 *Candida* isolates submitted for testing from 2016 through 2018, 43.9% were Candida albicans, 19.6% were Candida glabrata, 17.3% were Candida parapsilosis, 10.3% were Candida tropicalis, 4.9% were Candida dubliniensis, and 4.0% were Candida krusei (Table 1). Table 1 lists the frequencies of the most common species of *Candida* in each geographic region included in the SENTRY Program. C. albicans was most common in the Asia-Pacific (APAC) region (49.8%) and Europe (EUR) (49.6%) and least common in North America (NA; the United States and Canada; 34.1%), whereas C. glabrata was most common in NA (27.7%) and least common in Latin America (LATAM) (8.7%). C. parapsilosis and C. tropicalis were more common than C. glabrata in LATAM (20.2% and 20.2% versus 8.7%). C. tropicalis also was a frequent cause of IC in the APAC region (16.9%). C. krusei was more common in LATAM (6.2%), whereas C. dubliniensis was more common in NA (9.0%).

**TABLE 1:** Species distribution of *Candida* isolates by geographic region: SENTRY Program, 2016 to 2018

Region^*a*^	No. of isolates tested	% of isolates by species					
		C. albicans	C. glabrata	C. parapsilosis	C. tropicalis	C. dubliniensis	C. krusei
APAC	237	49.8	15.2	12.2	16.9	2.1	3.8
EUR	823	49.6	18.2	17.6	7.5	3.6	3.4
LATAM	242	43.0	8.7	20.2	20.2	1.7	6.2
NA	602	34.1	27.7	17.6	7.5	9.0	4.2
Total	1,904	43.9	19.6	17.3	10.3	4.9	4.0

a Abbreviations: APAC, Asia-Pacific; EUR, Europe; LATAM, Latin America; NA, North America.

### Rezafungin activity against *Candida*, Cryptococcus neoformans var. *grubii*, and *Aspergillus* isolates.

Among the 6 species of *Candida* for which the results are shown in Table 2, rezafungin was most active against C. albicans (MIC_90_, 0.06 μg/ml), C. tropicalis (MIC_90_, 0.06 μg/ml), and C. krusei (MIC_90_, 0.06 μg/ml) and least active against C. parapsilosis (MIC_90_, 2 μg/ml). With minimal variation over the 3-year time period, the modal MIC values were 0.03 μg/ml for C. albicans, C. tropicalis, and C. krusei, 0.06 μg/ml for C. glabrata and C. dubliniensis, and 1 μg/ml for C. parapsilosis. The MIC distribution data were employed to develop tentative epidemiological cutoff values (ECVs) using the iterative statistical method recommended by the CLSI (39) to establish the wild-type (WT) distribution for rezafungin and each of the tested species. The ECV of rezafungin for each species was 0.12 μg/ml for C. albicans (99.8% WT), C. glabrata (95.7% WT), C. tropicalis (97.4% WT), and C. krusei (100.0% WT), 0.25 μg/ml for C. dubliniensis (100.0% WT), and 4 μg/ml for C. parapsilosis (100.0% WT) (Table 2). Overall, 98.5% of the *Candida* spp. tested, aside from C. parapsilosis, were inhibited by ≤0.12 μg/ml and 99.2% were inhibited by ≤0.25 μg/ml of rezafungin (Table 2). Rezafungin showed limited activity against Cryptococcus neoformans (MIC_90_, >4 μg/ml) and was highly active against *Aspergillus* species (100% minimum effective concentration [MEC_100_], ≤0.03 μg/ml). The ECV calculated for Aspergillus fumigatus was 0.03 μg/ml.

**TABLE 2:** Antimicrobial activity of rezafungin tested against the main organisms and organism groups from all years using the CLSI method

Organism/organism group (no. of isolates)	No. (cumulative %) of isolates inhibited at MIC (μg/ml) of^*a*^:													MIC (μg/ml)	
	≤0.002	0.004	0.008	0.015	0.03	0.06	0.12	0.25	0.5	1	2	4	>^*b*^	50%	90%
Candida albicans (835)	11 (3.8)	6 (5.8)	87 (10.4)	270 (42.8)	309 (79.8)	139 (96.4)	28 (99.8)	2 (100.0)						0.03	0.06
2016 (276)			13 (4.7)	100 (40.9)	113 (81.9)	38 (95.7)	11 (99.6)	1 (100.0)						0.03	0.06
2017 (267)			12 (4.5)	83 (35.6)	96 (71.5)	66 (96.3)	10 (100.0)							0.03	0.06
2018 (292)	11 (3.8)	6 (5.8)	45 (21.2)	87 (51.0)	100 (85.3)	35 (97.3)	7 (99.7)	1 (100.0)						0.015	0.06
Candida glabrata (374)	1 (0.3)	0 (0.3)	0 (0.3)	5 (1.6)	136 (38.0)	162 (81.3)	54 (95.7)	6 (97.3)	3 (98.1)	3 (98.9)	4 (100.0)			0.06	0.12
2016 (135)			0 (0.0)	1 (0.7)	38 (28.9)	65 (77.0)	27 (97.0)	3 (99.3)	0 (99.3)	0 (99.3)	1 (100.0)			0.06	0.12
2017 (121)				0 (0.0)	33 (27.3)	60 (76.9)	20 (93.4)	2 (95.0)	3 (97.5)	2 (99.2)	1 (100.0)			0.06	0.12
2018 (118)	1 (0.8)	0 (0.8)	0 (0.8)	4 (4.2)	65 (59.3)	37 (90.7)	7 (96.6)	1 (97.5)	0 (97.5)	1 (98.3)	2 (100.0)			0.03	0.06
Candida parapsilosis (329)			0 (0.0)	1 (0.3)	0 (0.3)	0 (0.3)	1 (0.6)	5 (2.1)	62 (21.0)	134 (61.7	124 (99.4)	2 (100.0)		1	2
2016 (94)							0 (0.0)	2 (2.1)	13 (16.0)	37 (55.3)	42 (100.0)			1	2
2017 (118)			0 (0.0)	1 (0.8)	0 (0.8)	0 (0.8)	0 (0.8)	2 (2.5)	14 (14.4)	48 (55.1)	51 (98.3)	2 (100.0)		1	2
2018 (117)						0 (0.0)	1 (0.9)	1 (1.7)	35 (31.6)	49 (73.5)	31 (100.0)			1	2
Candida tropicalis (196)			12 (6.1)	52 (32.7)	75 (70.9)	41 (91.8)	11 (97.4)	3 (99.0)	0 (99.0)	1 (99.5)	1 (100.0)			0.03	0.06
2016 (64)			3 (4.7)	20 (35.9)	24 (73.4)	12 (92.2)	3 (96.9)	0 (96.9)	0 (96.9)	1 (98.4)	1 (100.0)			0.03	0.06
2017 (54)			0 (0.0)	11 (20.4)	19 (55.6)	17 (87.0)	5 (96.3)	2 (100.00)						0.03	0.12
2018 (78)		0 (0.0)	9 (11.5)	21 (38.5)	32 (79.5)	12 (94.9)	3 (98.7)	1 (100.0)						0.03	0.06
Candida krusei (77)			0 (0.0)	22 (28.6)	31 (68.8)	17 (90.9)	7 (100.0)							0.03	0.06
2016 (33)			0 (0.0)	8 (24.2)	17 (75.8)	7 (97.0)	1 (100.0)							0.03	0.06
2017 (28)			0 (0.0)	3 (10.7)	12 (53.6)	10 (89.3)	3 (100.0)							0.03	0.12
2018 (16)			0 (0.0)	11 (68.8)	2 (81.2)	0 (81.2)	3 (100.0)							0.015	0.12
Candida dubliniensis (93)	1 (1.1)	0 (1.1)	1 (2.2)	4 (6.5)	30 (38.7)	39 (80.6)	18 (100.0)							0.06	0.12
2016 (30)				0 (0.0)	8 (26.7)	15 (76.7)	7 (100.0)							0.06	0.12
2017 (28)			0 (0.0)	1 (3.6)	9 (35.7)	11 (75.0)	7 (100.0)							0.06	0.12
2018 (35)	1 (2.9)	0 (2.9)	1 (5.7)	3 (14.3)	13 (51.4)	13 (88.6)	4 (100.0)							0.03	0.12
Cryptococcus neoformans var. *grubii* (73)											0 (0.0)	9 (12.3)	64 (100.0)	>4	>4
2016 (27)												0 (0.0)	27 (100.0)	>4	>4
2017 (25)											0 (0.0)	7 (28.0)	18 (100.0)	>4	>4
2018 (21)											0 (0.0)	2 (9.5)	19 (100.0)	>4	>4
Aspergillus fumigatus (183)		3 (4.0)	64 (36.6)	88 (84.7)	28 (100.0)									0.015	0.03
2016 (48)			26 (54.2)	20 (95.8)	2 (100.0)									≤0.008	0.015
2017 (60)			25 (41.7)	29 (90.0)	6 (100.0)									0.015	0.015
2018 (75)	0 (0.0)	3 (4.0)	13 (21.3)	39 (73.3)	20 (100.0)									0.015	0.03
*Aspergillus* section *Flavi* (45)		5 (33.3)	20 (55.6)	18 (95.6)	2 (100.0)									≤0.008	0.015
2016 (12)			3 (25.0)	7 (83.3)	2 (100.0)									0.015	0.03
2017 (18)			8 (44.4)	10 (100.0)										0.015	0.015
2018 (15)	0 (0.0)	5 (33.3)	9 (93.3)	1 (100.0)										0.008	0.008

a During the 2016 and 2017 study years, the lowest echinocandins concentration tested was 0.008 μg/ml. The range was expanded to 0.002 μg/ml in 2018.

b >, greater than the last concentration tested.

### Rezafungin and comparator *in vitro* activity against *Candida*, C. neoformans var. *grubii*, and *Aspergillus* isolates.

Rezafungin (MIC_50/90_, 0.03/0.06 μg/ml; 99.8% WT) displayed activity against C. albicans comparable to that of anidulafungin, micafungin, and caspofungin (MIC_50/90_, 0.015/0.03 μg/ml [anidulafungin, caspofungin, and micafungin]) (Table 3). One C. albicans isolate was resistant (MIC, 1 μg/ml) to both caspofungin and micafungin and non-wild type (NWT [in which the MIC is greater than the ECV]; 0.25 μg/ml) to rezafungin while harboring a mutation in *fks1* hot spot (HS) region 1 (HS1; S645P) (Table 4). Three fluconazole-resistant strains were detected; one was from LATAM, and two were from NA (Table 5).

**TABLE 3:** Antimicrobial activity of rezafungin and comparator agents tested against fungal isolates from the worldwide 2016 to 2018 rezafungin surveillance program^*a*^

Antimicrobial agent	MIC (μg/ml)		CLSI^*b*^		ECV^*b*^	
	50%	90%	% S	% R	% WT	% NWT
Candida albicans (*n* = 835)						
Rezafungin	0.03	0.06			99.8	0.2
Anidulafungin	0.015	0.03	100.0	0.0	100.0	0.0
Caspofungin	0.015	0.03	99.9	0.1		
Micafungin	0.015	0.03	99.9	0.1	99.6	0.4
Fluconazole	≤0.12	0.25	99.5	0.4	98.1	1.9
Itraconazole	≤0.06	0.12				
Posaconazole	0.03	0.06			96.5	3.5
Voriconazole	≤0.008	0.015	99.9	0.0	99.0	1.0
Amphotericin B	0.5	1			100.0	0.0
Candida glabrata (*n* = 374)						
Rezafungin	0.06	0.12			95.7	4.3
Anidulafungin	0.06	0.12	94.4	3.2	96.8	3.2
Caspofungin	0.03	0.06	97.1	2.1		
Micafungin	0.015	0.03	96.0	2.4	93.3	6.7
Fluconazole	2	32	91.4^*c*^	8.6	85.6	14.4
Itraconazole	0.5	2			98.7	1.3
Posaconazole	0.25	1			93.0	7.0
Voriconazole	0.06	1			87.2	12.8
Amphotericin B	1	1			100.0	0.0
Candida parapsilosis (*n* = 329)						
Rezafungin	1	2			100.0	0.0
Anidulafungin	2	2	93.9	0.0	100.0	0.0
Caspofungin	0.25	0.5	100.0	0.0		
Micafungin	1	1	100.0	0.0	100.0	0.0
Fluconazole	0.5	32	86.0	12.5	83.6	16.4
Itraconazole	0.12	0.25				
Posaconazole	0.06	0.12			100.0	0.0
Voriconazole	≤0.008	0.25	88.4	0.9	84.5	15.5
Amphotericin B	0.5	1			100.0	0.0
Candida tropicalis (*n* = 196)						
Rezafungin	0.03	0.06			100.0	0.0
Anidulafungin	0.03	0.06	99.0	1.0	98.0	2.0
Caspofungin	0.015	0.06	99.0	1.0		
Micafungin	0.03	0.06	99.0	1.0	96.4	3.6
Fluconazole	0.25	1	96.9	2.6	94.9	5.1
Itraconazole	0.12	0.5			100.0	0.0
Posaconazole	0.06	0.12			92.9	7.1
Voriconazole	0.015	0.06	96.9	0.0	96.9	3.1
Amphotericin B	0.5	1			100.0	0.0
Candida krusei (*n* = 77)						
Rezafungin	0.03	0.06			100.0	0.0
Anidulafungin	0.06	0.12	100.0	0.0	100.0	0.0
Caspofungin	0.12	0.25	98.7	0.0		
Micafungin	0.06	0.12	100.0	0.0	100.0	0.0
Fluconazole	32	64				
Itraconazole	0.5	1			100.0	0.0
Posaconazole	0.5	0.5			100.0	0.0
Voriconazole	0.25	0.5	96.1	1.3	96.1	3.9
Amphotericin B	1	2			100.0	0.0
Candida dubliniensis (*n* = 93)						
Rezafungin	0.06	0.12			100.0	0.0
Anidulafungin	0.03	0.12			100.0	0.0
Caspofungin	0.03	0.03				
Micafungin	0.03	0.03			100.0	0.0
Fluconazole	≤0.12	0.25			96.8	3.2
Itraconazole	≤0.06	0.25				
Posaconazole	0.03	0.06				
Voriconazole	≤0.008	0.015				
Amphotericin B	0.5	0.5				
Cryptococcus neoformans var. *grubii* (*n* = 73)						
Rezafungin	>4	>4				
Anidulafungin	>4	>4				
Caspofungin	>4	>4				
Micafungin	>4	>4				
Fluconazole	2	4			100.0	0.0
Itraconazole	0.25	0.25			93.5	6.5
Posaconazole	0.12	0.25			97.3	2.7
Voriconazole	0.03	0.12			100.0	0.0
Amphotericin B	0.5	1			52.1	47.9
Aspergillus fumigatus (*n* = 183)						
Rezafungin	0.015	0.03			100.0	0.0
Anidulafungin	0.015	0.03				
Caspofungin	0.015	0.03			100.0	0.0
Micafungin	≤0.008	0.015				
Itraconazole	0.5	1			98.4	1.6
Posaconazole	0.25	0.5				
Voriconazole	0.25	0.5			98.9	1.1
Amphotericin B	1	2			100.0	0.0
*Aspergillus* section *Flavi* (*n* = 45)						
Rezafungin	≤0.008	0.015				
Anidulafungin	≤0.008	0.015				
Caspofungin	0.015	0.03			100.0	0.0
Micafungin	0.015	0.03				
Itraconazole	0.5	1			100.0	0.0
Posaconazole	0.25	0.5			100.0	0.0
Voriconazole	0.5	1			100.0	0.0
Amphotericin B	2	2			100.0	0.0

a Abbreviations: S, susceptible; R, resistant; WT, wild type; NWT, non-wild type.

b Criteria were published in the CLSI M60 document (40). Epidemiological cutoff value (ECV) criteria were published in the CLSI M59 document (41). The ECVs for rezafungin and each species were determined from data in the present study.

c Nonresistant is interpreted as susceptible-dose dependent.

**TABLE 4:** Summary of *fks* alterations detected in *Candida* strains as part of the 2016 to 2018 rezafungin surveillance program^*a*^

Isolate	Country	Yr	Organism	MIC (μg/ml) by CLSI method				1,3-β-d-Glucan synthase mutation in:			
				RFG	AFG	CAS	MFG	*fks1* HS1	*fks1* HS2	*fks2* HS1	*fks2* HS2
1051621	Hungary	2018	C. tropicalis	0.25	0.25	0.12	0.12	WT	WT	NT	NT
1051641	Hungary	2018	C. glabrata	1	1	1	0.5	WT	WT	F659_del	WT
1053234	Canada	2018	C. glabrata	0.12	0.12	0.06	0.06	WT	WT	WT	WT
1075570	Belgium	2018	C. glabrata	0.06	0.12	0.06	0.06	WT	WT	WT	WT
1078854	USA	2018	C. glabrata	0.06	0.06	0.06	0.06	WT	WT	F659_del	WT
1078861	USA	2018	C. glabrata	2	2	1	1	WT	WT	S663P	WT
1085740	Spain	2018	C. tropicalis	0.06	0.06	0.06	0.12	WT	WT	NT	NT
1087598	USA	2018	C. glabrata	2	4	4	4	WT	WT	S663P	WT
997524	Mexico	2017	C. glabrata	0.5	0.5	0.25	0.06	F625S	WT	WT	WT
999721	Italy	2017	C. glabrata	0.06	0.06	0.06	0.06	WT	WT	WT	WT
1015009	Spain	2017	C. glabrata	0.5	1	0.5	0.25	WT	WT	Y657 deletion, F658Y	WT
1020535	USA	2017	C. glabrata	0.25	0.25	0.12	0.06	WT	WT	WT	WT
1021070	France	2017	C. glabrata	1	2	0.5	0.5	WT	WT	S663P	WT
1025460	USA	2017	C. glabrata	0.5	1	0.5	1	S629P	WT	R665G	WT
1026179	Spain	2017	C. glabrata	1	1	0.25	0.25	WT	WT	Y657 deletion, F658Y	WT
1034513	Ireland	2017	C. glabrata	2	4	2	0.5	WT	WT	S663P	WT
1034514	Ireland	2017	C. glabrata	0.25	0.5	0.12	0.12	WT	WT	S663P	WT
1034803	USA	2017	C. glabrata	0.12	0.12	0.06	0.06	WT	WT	WT	WT
1034763	Turkey	2017	C. tropicalis	0.06	0.06	0.25	0.12	WT	WT	NT	NT
1034766	Turkey	2017	C. tropicalis	0.12	0.25	0.03	0.06	WT	WT	NT	NT
1041544	Greece	2017	C. tropicalis	0.06	0.06	0.03	0.12	WT	WT	NT	NT
984357	Ireland	2016	C. albicans	0.25	0.12	1	1	S645P	WT	NT	NT
978825	Turkey	2016	C. albicans	0.12	0.12	0.12	0.06	WT	WT	NT	NT
948247	USA	2016	C. glabrata	0.06	0.12	0.03	0.03	WT	WT	WT	WT
949151	USA	2016	C. glabrata	0.03	0.06	0.06	0.12	WT	WT	WT	WT
970382	USA	2016	C. glabrata	0.25	0.25	0.12	0.12	S629P	WT	WT	WT
970397	USA	2016	C. glabrata	0.12	0.25	0.25	0.12	WT	WT	P667H	WT
974239	USA	2016	C. glabrata	0.25	0.25	0.06	0.12	S629P	WT	WT	WT
974249	USA	2016	C. glabrata	2	2	1	1	WT	WT	S663P	WT
978819	Turkey	2016	C. glabrata	0.25	0.25	0.06	0.06	WT	WT	WT	WT
983007	USA	2016	C. glabrata	0.12	0.5	0.06	0.12	WT	WT	F658_del	WT
985673	USA	2016	C. glabrata	0.06	0.12	0.06	0.06	WT	WT	S663P	WT
936285	Germany	2016	C. krusei	0.12	0.12	0.25	0.12	WT	WT	NT	NT
954660	Italy	2016	C. krusei	0.015	0.03	0.06	0.06	WT	WT	NT	NT
975699	USA	2016	C. krusei	0.015	0.06	0.12	0.06	WT	WT	NT	NT
977046	Brazil	2016	C. krusei	0.015	0.015	0.06	0.06	WT	WT	NT	NT
970388	USA	2016	C. tropicalis	2	1	>8	2	S654P	WT	NT	NT
977041	Brazil	2016	C. tropicalis	1	1	2	1	F650S	WT	NT	NT

a RFG, rezafungin; AFG, anidulafungin; CAS, caspofungin; MFG, micafungin; WT, wild type; NT, not tested.

**TABLE 5:** Fluconazole resistance by geographic region for *Candida* species, SENTRY Program, 2016 to 2018^*a*^

Species	Region	No. of isolates tested	% (no.) of isolates resistant
C. albicans	APAC	118	0.0 (0)
	EUR	408	0.0 (0)
	LATAM	104	1.0 (1)
	NA	205	1.0 (2)
	Total	835	0.4 (3)
C. glabrata	APAC	36	2.8 (1)
	EUR	150	6.0 (9)
	LATAM	21	0.0 (0)
	NA	167	13.2 (22)
	Total	374	8.6 (32)
C. parapsilosis	APAC	29	3.4 (1)
	EUR	145	24.8 (36)
	LATAM	49	0.0 (0)
	NA	106	3.8 (4)
	Total	329	12.5 (41)
C. tropicalis	APAC	40	5.0 (2)
	EUR	62	1.6 (1)
	LATAM	49	4.1 (2)
	NA	45	0.0 (0)
	Total	196	2.6 (5)
C. dubliniensis^*b*^	APAC	5	0.0 (0)
	EUR	30	0.0 (0)
	LATAM	4	0.0 (0)
	NA	54	5.6 (3)
	Total	93	3.2 (3)

a APAC, Asia-Pacific; EUR, Europe; LATAM, Latin America; NA, North America.

b Percentage of wild-type isolates based on epidemiological cutoff value (ECV) criteria published in the CLSI M59 document (41).

Among the 374 C. glabrata isolates tested, 95.7% were inhibited by rezafungin (MIC_50/90_, 0.06/0.12 μg/ml) at the ECV cutoff value of ≤0.12 μg/ml (Tables 2 and 3). Micafungin (MIC_50/90_, 0.015/0.03 μg/ml), caspofungin (MIC_50/90_, 0.03/0.06 μg/ml), and anidulafungin (MIC_50/90_, 0.06/0.12 μg/ml), respectively, inhibited 96.0%, 97.1%, and 94.4% of these isolates at the current CLSI breakpoints (40). Mutations within the *fks* HS leading to amino acid alterations were found in 17 (68.0%) out of 25 C. glabrata isolates displaying echinocandin MIC values greater than the ECV (Table 4). The most common substitutions were *fks2* HS1 S663P (7 isolates), *fks2* HS1 with a deletion of F659 (F659_del) (2 isolates), *fks2* HS1 Y657_del/F658Y (2 isolates), and *fks1* HS1 S629P (2 isolates). The corresponding rezafungin MIC values ranged from 0.06 to 2 μg/ml (82.4% > ECV [0.12 μg/ml]) for all 17 isolates with an *fks* mutation (Table 4). Among all C. glabrata isolates collected from 2016 to 2018, 8.6% displayed a fluconazole-resistant phenotype. Based on the ECV cutoff published by CLSI, 7.0% and 12.8% of these isolates were categorized as NWT to posaconazole and voriconazole, respectively (40, 41) (Table 3). High rates of resistance to fluconazole were seen in isolates from EUR (6.0%) and NA (13.2%) (Table 5). Not only was C. glabrata a rare cause of IC in LATAM (Table 1), but also it was less resistant to fluconazole (0.0%) than isolates from the other monitored regions (Table 5).

Rezafungin inhibited all C. parapsilosis isolates (*n* = 329) at the ECV of ≤4 μg/ml (Table 2). The activity of rezafungin (MIC_90_, 2 μg/ml) was similar to that observed for micafungin (MIC_90_, 1 μg/ml; 100.0% S) and anidulafungin (MIC_90_, 2 μg/ml; 93.9% S) and was 4-fold lower than that of caspofungin (MIC_90_, 0.5 μg/ml; 100.0% S) (Tables 2 and 3). Among the C. parapsilosis isolates, a total of 41 isolates (12.5%) were categorized as fluconazole resistant, and 36 of these strains (87.8%) were from European medical centers (24.8% fluconazole resistant) (Table 5). Although C. parapsilosis was common in LATAM (20.2% of *Candida* isolates, second in rank order) (Table 1), no fluconazole-resistant strains were detected among the 49 C. parapsilosis isolates tested (Table 5).

C. tropicalis isolates (*n* = 196) were largely susceptible to anidulafungin, caspofungin, and micafungin (99.0% S) (Table 3). Rezafungin (MIC_50/90_, 0.03/0.06 μg/ml) inhibited 97.4% of the isolates at the proposed ECV of ≤0.12 μg/ml (Tables 2 and 3). Among 7 C. tropicalis isolates categorized as NWT to echinocandin and submitted to *fks* sequencing, 2 harbored *fks1* HS1 mutations leading to amino acid alterations (S645P and F650S; Table 4). Both isolates were resistant to anidulafungin (MIC values, 1 μg/ml for both), caspofungin (MIC values, >8 and 2 μg/ml), and micafungin (MIC values, 2 and 1 μg/ml) and NWT (MIC > ECV; MIC values, 2 and 1 μg/ml) to rezafungin. The remaining 5 isolates did not contain *fks1* mutations, and 4 were WT to rezafungin (MIC values, ≤0.12 μg/ml). Fluconazole resistance was observed in 5 C. tropicalis isolates (2.6% of the total; Table 5). No fluconazole-resistant strains were found among the 45 C. tropicalis isolates from NA, and 5.0% of the isolates from APAC were resistant to fluconazole (Table 5).

Rezafungin (MIC_50/90_, 0.03/0.06 μg/ml) was active against 77 C. krusei isolates; 100.0% of the isolates were inhibited at ≤0.12 μg/ml, the ECV for this species (100.0% WT) (Tables 2 and 3). These isolates were susceptible to anidulafungin (MIC_50/90_, 0.06/0.12 μg/ml; 100.0% S), micafungin (MIC_50/90_, 0.06/0.12 μg/ml; 100.0% S), and caspofungin (MIC_50/90_, 0.12/0.25 μg/ml; 98.7% S) (Table 3) according to CLSI breakpoint criteria. Four C. krusei isolates were NWT to one or more echinocandins, but none of these isolates were shown to possess an *fks* mutation: all were WT to rezafungin (Table 4). Voriconazole was active against 96.1% of the C. krusei isolates, and all isolates displayed a posaconazole WT phenotype (Table 3).

All echinocandins (anidulafungin [MIC_50/90_, 0.03/0.12 μg/ml; 100.0% WT], caspofungin [MIC_50/90_, 0.03/0.03 μg/ml], and micafungin [MIC_50/90_, 0.03/0.03 μg/ml; 100.0% WT]) displayed activity similar to that of rezafungin (MIC_50/90_, 0.06/0.12 μg/ml; 100.0% WT) (Tables 2 and 3) against 93 C. dubliniensis isolates. Three isolates were resistant/NWT to fluconazole, and all three were from patients hospitalized in NA (5.6% resistant) (Table 5).

Fluconazole (MIC_50/90_, 2/4 μg/ml) and other azoles (MIC_50/90_ values, 0.12/0.25 and 0.03/0.12 μg/ml for posaconazole and voriconazole, respectively) displayed good activity against C. neoformans, whereas echinocandins, including rezafungin, displayed limited activity.

The activity of rezafungin against 183 A. fumigatus isolates tested (MEC_50/90_, 0.015/0.03 μg/ml; all were inhibited at the ECV of ≤0.03 μg/ml [100.0% WT]) was comparable to that of caspofungin (MEC_50/90_, 0.015/0.03 μg/ml), anidulafungin (MEC_50/90_, 0.015/0.03 μg/ml; 100% WT), and micafungin (MEC_50/90_, ≤0.008/0.015 μg/ml). Voriconazole and itraconazole showed WT MIC values against over 98% of the A. fumigatus isolates (Table 3).

Against Aspergillus flavus species complex isolates (*n* = 45), comparable activity was observed for rezafungin (MEC_50/90_, ≤0.008/0.015 μg/ml) and the other echinocandins, such as caspofungin (MEC_50/90_, 0.015/0.03 μg/ml; 100% WT), anidulafungin (MEC_50/90_, ≤0.008/0.015 μg/ml), and micafungin (0.015/0.03 μg/ml). A WT phenotype was observed for itraconazole, posaconazole, and voriconazole against all A. flavus species complex isolates (Table 3).

## DISCUSSION

This study provides a robust estimate of the WT MIC/MEC distributions of rezafungin for 6 species of *Candida* as well as A. fumigatus and A. flavus and expands upon our earlier rezafungin activity observations (31–33). Although establishing definitive ECVs and clinical breakpoints (CBPs) for rezafungin requires multicenter studies involving larger numbers of isolates of each species than the numbers used in this study (39), we suggest that the ECV determined using CLSI BMD methods in the present study is ≤0.12 μg/ml for C. albicans, C. tropicalis, C. glabrata, and C. krusei (98.5% of 1,482 isolates; Table 2), ≤0.25 μg/ml for C. dubliniensis (100.0% of 93 isolates), ≤4 μg/ml for C. parapsilosis (100.0% of 329 isolates), and ≤0.03 μg/ml for A. fumigatus (100.0% of 183 isolates) (Table 2). Notably, these values are far below the peak plasma concentrations of 22 to 30 μg/ml achievable at the 400-mg dose (15, 26, 27) and are equivalent to the ECVs established for these species/species groups and the clinically available echinocandins (41–43).

Additional support for these ECVs is found in a recent multicenter study of rezafungin activity against *Candida* spp. determined using the EUCAST BMD method and both visual and statistical means of determining possible wild-type upper-limit (WT-UL) values (28). In the four-laboratory study, WT-UL cutoffs were proposed for C. glabrata (0.125 μg/ml), C. krusei (0.125 μg/ml), and C. parapsilosis (4 μg/ml). Although interlaboratory variation precluded proposing cutoffs for C. albicans and C. tropicalis, the WT-UL statistical 97.5% endpoint was 0.063 μg/ml for C. albicans and 0.25 μg/ml for C. tropicalis (28). These values compare favorably with the ECVs generated by the CLSI BMD method in the present study. Although an essential agreement rate (±2 dilution steps) of 92.0% for C. albicans and 100.0% for C. glabrata, C. parapsilosis, C. tropicalis, and C. krusei between CLSI and EUCAST methods for rezafungin was observed previously (31), alignment between CLSI and EUCAST susceptibility profiles and breakpoints is yet to be determined, as significant interlaboratory EUCAST MIC variability (likely attributed to nonspecific binding of the drug to plastics) has been identified for rezafungin against a more susceptible collection of *Candida* spp. (28, 44).

As seen in Table 4, the highest rezafungin MIC values for *fks* mutant strains of C. albicans and C. glabrata were 0.25 μg/ml and 2 μg/ml, respectively. Both of these MIC values for mutant strains are within the range of concentrations that Bader et al. (20) estimated would achieve percent probabilities of PK-PD target attainment of 100% through week 6, suggesting that weekly regimens of rezafungin can achieve exposures associated with efficacy against some *fks* mutant *Candida* isolates (20). In addition, the same study showed that the mutant prevention concentration, the concentration of drug that would inhibit emerging resistant mutants, for both rezafungin and micafungin was 16 μg/ml (27). Given that the high plasma drug exposure of rezafungin easily exceeds the mutant prevention concentration for *Candida*, a possible advantage of rezafungin may be to prevent the development of resistance to the echinocandin class of antifungal agents (20, 22, 24, 27).

Expert panel guidelines from both NA (5) and EUR (12) favor step-down therapy to fluconazole or voriconazole for patients with candidiasis in specific clinical situations, that is, when clinical improvement and the clearance of *Candida* from the bloodstream are achieved by the initial echinocandin therapy. In addition, the organism must be susceptible to fluconazole (e.g., C. albicans, C. parapsilosis, and C. tropicalis) or voriconazole (e.g., C. krusei). Unfortunately, antifungal susceptibility testing is still not routinely available in many patient care settings. In these circumstances, clinicians are forced to rely on simple identification of the *Candida* species as a predictor of fluconazole susceptibility (5, 12). In most instances, isolates of C. albicans, C. parapsilosis, and C. tropicalis are considered to be reliably susceptible to fluconazole (16), whereas C. glabrata and C. krusei are considered to be intrinsically less susceptible or resistant and are suboptimal targets for using fluconazole (5, 12). This approach may be seriously flawed if fluconazole resistance emerges among the traditionally susceptible species. Concern regarding this approach has been raised by Oxman et al. (45), who found that despite the small proportion of C. albicans, C. parapsilosis, and C. tropicalis isolates with resistance/decreased susceptibility to fluconazole, these species comprised 36% of the reduced-susceptibility group (including C. glabrata and C. krusei), potentially compromising therapy with the resultant clinical failure. These concerns are supported by data from the current survey showing that the rate of resistance to fluconazole was 0.4% for C. albicans, 12.5% for C. parapsilosis, and 2.6% for C. tropicalis (Tables 3 and 5). In aggregate, these three normally susceptible species account for 31% of all fluconazole-resistant isolates. Species identification should be used cautiously as the sole criterion for anti-*Candida* agent selection (5, 45).

The increased rate of fluconazole resistance among the C. parapsilosis (12.5% overall) and C. tropicalis (2.6% overall) isolates in the present study is important, as these species are the non-C. albicans species most commonly isolated in LATAM (Table 1). Although less common than C. glabrata in EUR, the rate of fluconazole resistance of 24.8% among C. parapsilosis isolates exceeds the rate observed among C. glabrata (6.0%) isolates and is cause for alarm (Table 5).

This survey has some limitations, as noted elsewhere (16): the SENTRY Surveillance Program is a sentinel surveillance and is not population based; therefore, we may over- or underestimate the activity of the tested agents. In addition, we do not collect data concerning antifungal use or outcomes of therapy. The purpose of the SENTRY Program is to identify trends in antifungal resistance and to document the emergence of new species as well as the activity of new and established agents against key fungal pathogens. The broad geographic distribution, the longitudinal nature of the surveillance, and the use of molecular and proteomic identification methods and determination of resistance mechanisms are strengths of the SENTRY Program.

In conclusion, we have provided additional *in vitro* data demonstrating the activity of rezafungin against a collection of largely echinocandin-WT isolates of *Candida* spp., C. neoformans, A. fumigatus, and the A. flavus species complex. Given these findings, we suggest that MIC values of ≤0.12 μg/ml (C. albicans, C. glabrata, C. tropicalis, and C. krusei), ≤0.25 μg/ml (C. dubliniensis), and ≤4 μg/ml (C. parapsilosis) and a MEC of ≤0.03 μg/ml (A. fumigatus) approximate the ECV/WT-UL MIC/MEC distributions for rezafungin and the common species of *Candida* and *Aspergillus*. Further evaluations including at least 100 MIC values per species tested by three different laboratories should be performed to define the ECVs for rezafungin, a fundamental step in establishing clinical breakpoints.

This survey provides new information regarding emerging fluconazole resistance among C. parapsilosis and C. tropicalis clinical isolates from geographic regions beyond NA, in addition to demonstrating evidence of the sustained activity of rezafungin and the other echinocandins against *Candida* and *Aspergillus* species. Whereas the highest rates of fluconazole resistance in NA isolates were seen in C. glabrata (13.2%), fluconazole-resistant C. parapsilosis (24.8%) was most prominent in EUR and fluconazole-resistant C. tropicalis was most prominent in APAC (5.0%) and LATAM (4.1%). In all three instances, the rate of fluconazole resistance was highest in species of *Candida* other than C. glabrata. Species identification should be used cautiously as the sole criterion for selecting antifungal therapy.

## MATERIALS AND METHODS

### Organisms.

During 2016 to 2018, 2,205 nonduplicate fungal isolates were prospectively collected from 57 medical centers located in North America (723 isolates; 18 sites [17 sites in the United States and 1 site in Canada]), EUR (927 isolates; 22 sites, 14 countries), the APAC region (279 isolates; 11 sites, 5 countries), and LATAM (276 isolates; 6 sites, 4 countries). Isolates were recovered from the following sources: bloodstream infections (*n* = 1,460 isolates), pneumonia in hospitalized patients (*n* = 306), intra-abdominal infections (*n* = 32), skin and skin structure infections (*n* = 106), urinary tract infections (*n* = 35), and other or nonspecified body sites (*n* = 266).

### Fungal identification methods.

Yeast isolates were subcultured and screened using CHROMagar *Candida* (Becton, Dickinson, Sparks, MD) to ensure purity. Matrix-assisted laser desorption ionization–time of flight mass spectrometry (MALDI-TOF MS) was applied for the identification of all yeast isolates using a MALDI Biotyper apparatus according to the manufacturer’s instructions (Bruker Daltonics, Billerica, MA). Isolates that were not identified by proteomic methods were submitted to the previously described sequencing-based methods (43, 46, 47).

Molds were cultured and identified by MALDI-TOF MS or DNA sequencing analysis when an acceptable identification was not achieved by MALDI-TOF MS. The sequences of the 28S ribosomal DNA and β-tubulin genes of *Aspergillus* spp. were analyzed (47–50).

Nucleotide sequences were analyzed using Lasergene software (DNAStar, Madison, WI, USA) and compared to available sequences using the BLAST program (https://blast.ncbi.nlm.nih.gov/Blast.cgi).

### Antifungal susceptibility testing.

All isolates were tested by CLSI BMD methods as described in documents M27 (37) and M38 (38). Only systemically active antifungal agents were tested, including rezafungin, anidulafungin, micafungin, caspofungin, itraconazole, fluconazole, voriconazole, posaconazole, and amphotericin B. The ranges of antifungal agent concentrations tested were 0.008 to 4 μg/ml for itraconazole, posaconazole, and voriconazole, 0.12 to 2 μg/ml for amphotericin B, and 0.12 to 128 μg/ml for fluconazole. The echinocandin concentration range tested during 2016 and 2017 was 0.008 to 4 μg/ml, whereas this range was expanded to 0.002 to 4 μg/ml in 2018. MIC results were determined visually after 24 h (*Candida* spp.), 48 h (*Aspergillus* spp.), or 72 h (C. neoformans) of incubation at 35°C. Azole and echinocandin MIC values against yeasts were read as the lowest concentration of drug that resulted in ≥50% inhibition of growth relative to that of the growth control. Complete (100%) inhibition was used to determine itraconazole, posaconazole, and voriconazole MIC values against *Aspergillus* spp. and amphotericin B MIC values against yeasts and molds. Echinocandin minimum effective concentration (MEC) values, including those of rezafungin, were read against *Aspergillus* spp. as described in CLSI document M38 (38).

Echinocandin, fluconazole, and voriconazole susceptibility categories were applied for the five most common species of *Candida* (C. albicans, C. tropicalis, C. parapsilosis, C. glabrata, and C. krusei) following CLSI clinical breakpoints (CBPs) (40). Epidemiological cutoff values (ECVs/ECOFFs) were used to differentiate wild-type (WT) from non-wild-type (NWT) isolates of the species for which there are no CLSI CBPs (39, 41). Neither CBPs nor ECVs/ECOFFs have been determined by CLSI methods for rezafungin against *Candida*, *Aspergillus*, or *Cryptococcus* spp. For comparison, we established tentative ECVs for rezafungin and each species using the iterative statistical method recommended by CLSI (28, 32, 39–41). These ECVs must be considered tentative, given the CLSI requirement that ECVs be determined using MIC/MEC data acquired from a minimum of three different laboratories including at least 100 MIC/MEC values from 100 individual isolates, all determined by CLSI reference methods (39).

### QC.

To ensure proper test conditions and procedures, concurrent quality control (QC) testing was performed. The QC strains recommended by CLSI included C. krusei ATCC 6258, C. parapsilosis ATCC 22019, A. flavus ATCC 204304, and A. fumigatus ATCC MYA-3626.

### Screening for 1,3-β-d-glucan synthase mutations.

All *Candida* isolates that were echinocandin resistant or that showed MIC values higher than the ECV for any echinocandin were submitted to whole-genome sequencing for detecting mutations in the HS regions of *fks1* and *fks2* (C. glabrata only) as described previously (43, 48, 50).

## References

[1] Bassetti M, Righi E, Montravers P, Cornely OA 2018 What has changed in the treatment of invasive candidiasis? A look at the past 10 years and ahead. J Antimicrob Chemother 73:i14–i25. doi:10.1093/jac/dkx445. 29304208 PMC5890781

[2] Berkow EL, Lockhart SR 2017 Fluconazole resistance in *Candida* species: a current perspective. Infect Drug Resist 10:237–245. doi:10.2147/IDR.S118892. 28814889 PMC5546770

[3] Lockhart SR, Berkow EL, 2019 Antifungal agents, p 2319–2333. *In* Carroll KC, Pfaller MA, Landry ML, McAdam AJ, Patel R, Richter SS, Warnock DW (ed), Manual of clinical microbiology, 12th ed, vol 2 ASM Press, Washington, DC.

[4] Pakyz AL, Gurgle HE, Oinonen MJ 2011 Antifungal use in hospitalized adults in U.S. academic health centers. Am J Health Syst Pharm 68:415–418. doi:10.2146/ajhp100423. 21330683

[5] Pappas PG, Kauffman CA, Andes D, Clancy CJ, Marr KA, Ostrosky-Zeichner L, Reboli AC, Schuster MG, Vazquez JA, Walsh TJ, Zaoutis TE, Sobel JD 2016 Clinical practice guideline for the management of candidiasis: 2016 update by the Infectious Diseases Society of America. Clin Infect Dis 62:409–417. doi:10.1093/cid/civ1194. 26679628 PMC4725385

[6] Patterson TF, Thompson GR III, Denning DW, Fishman JA, Hadley S, Herbrecht R, Kontoyiannis DP, Marr KA, Morrison VA, Nguyen MH, Segal BH, Steinbach WJ, Stevens DA, Walsh TJ, Wingard JR, Young JA, Bennett JE 2016 Practice guidelines for the diagnosis and management of aspergillosis: 2016 update by the Infectious Diseases Society of America. Clin Infect Dis 63:e1–e60. doi:10.1093/cid/ciw326. 27365388 PMC4967602

[7] Vallabhaneni S, Baggs J, Tsay S, Srinivasan AR, Jernigan JA, Jackson BR 2018 Trends in antifungal use in US hospitals, 2006-12. J Antimicrob Chemother 73:2867–2875. doi:10.1093/jac/dky270. 30295769

[8] Fondevilla E, Grau S, Mojal S, Palomar M, Matas L, Gudiol F, VINCat Group 2016 Consumption of systemic antifungal agents among acute care hospitals in Catalonia (Spain), 2008-2013. Expert Rev Anti Infect Ther 14:137–144. doi:10.1586/14787210.2016.1096776. 26466197

[9] Garey KW, Aitken SL, Dima-Ala A, Beyda ND, Kuper K, Xie Y, Koo HL 2015 Echinocandin use in hospitalized patients: a multi-institutional study. Am J Med Sci 349:316–320. doi:10.1097/MAJ.0000000000000412. 25607510

[10] Goemaere B, Lagrou K, Spriet I, Hendrickx M, Vandael E, Becker P, Catry B 2019 Systemic antifungal drug use in Belgium—one of the biggest antifungal consumers in Europe. Mycoses 62:542–550. doi:10.1111/myc.12912. 30887582

[11] Stultz JS, Kohinke R, Pakyz AL 2018 Variability in antifungal utilization among neonatal, pediatric, and adult inpatients in academic medical centers throughout the United States of America. BMC Infect Dis 18:501. doi:10.1186/s12879-018-3410-4. 30285738 PMC6171307

[12] Cornely OA, Bassetti M, Calandra T, Garbino J, Kullberg BJ, Lortholary O, Meersseman W, Akova M, Arendrup MC, Arikan-Akdagli S, Bille J, Castagnola E, Cuenca-Estrella M, Donnelly JP, Groll AH, Herbrecht R, Hope WW, Jensen HE, Lass-Florl C, Petrikkos G, Richardson MD, Roilides E, Verweij PE, Viscoli C, Ullmann AJ, ESCMID Fungal Infection Study Group 2012 ESCMID guideline for the diagnosis and management of *Candida* diseases 2012: non-neutropenic adult patients. Clin Microbiol Infect 18(Suppl 7):19–37. doi:10.1111/1469-0691.12039. 23137135

[13] Andes DR, Safdar N, Baddley JW, Playford G, Reboli AC, Rex JH, Sobel JD, Pappas PG, Kullberg BJ, Mycoses Study Group 2012 Impact of treatment strategy on outcomes in patients with candidemia and other forms of invasive candidiasis: a patient-level quantitative review of randomized trials. Clin Infect Dis 54:1110–1122. doi:10.1093/cid/cis021. 22412055

[14] Lamoth F, Lockhart SR, Berkow EL, Calandra T 2018 Changes in the epidemiological landscape of invasive candidiasis. J Antimicrob Chemother 73:i4–i13. doi:10.1093/jac/dkx444. 29304207 PMC11931512

[15] Sofjan AK, Mitchell A, Shah DN, Nguyen T, Sim M, Trojcak A, Beyda ND, Garey KW 2018 Rezafungin (CD101), a next-generation echinocandin: a systematic literature review and assessment of possible place in therapy. J Glob Antimicrob Resist 14:58–64. doi:10.1016/j.jgar.2018.02.013. 29486356

[16] Pfaller MA, Diekema DJ, Turnidge JD, Castanheira M, Jones RN 2019 Twenty years of the SENTRY Antifungal Surveillance Program: results for Candida species from 1997-2016. Open Forum Infect Dis 6:S79–S94. doi:10.1093/ofid/ofy358. 30895218 PMC6419901

[17] Beyda ND, Lewis RE, Garey KW 2012 Echinocandin resistance in *Candida* species: mechanisms of reduced susceptibility and therapeutic approaches. Ann Pharmacother 46:1086–1096. doi:10.1345/aph.1R020. 22811350

[18] Yamaguchi M, Kurokawa T, Ishiyama K, Aoki G, Ueda M, Matano S, Takami A, Yamazaki H, Sawazaki A, Yamauchi H, Yoshida T, Nakao S 2011 Efficacy and safety of micafungin as an empirical therapy for invasive fungal infections in patients with hematologic disorders: a multicenter, prospective study. Ann Hematol 90:1209–1217. doi:10.1007/s00277-011-1277-1. 21695388

[19] Yamazaki S, Nakamura F, Yoshimi A, Ichikawa M, Nannya Y, Kurokawa M 2014 Safety of high-dose micafungin for patients with hematological diseases. Leuk Lymphoma 55:2572–2576. doi:10.3109/10428194.2014.885514. 24460099

[20] Bader JC, Lakota EA, Flanagan S, Ong V, Sandison T, Rubino CM, Bhavnani SM, Ambrose PG 2018 Overcoming the resistance hurdle: pharmacokinetic-pharmacodynamic target attainment analyses for rezafungin (CD101) against *Candida albicans* and *Candida glabrata*. Antimicrob Agents Chemother 62:e02614-17. doi:10.1128/AAC.02614-17. 29555634 PMC5971579

[21] James KD, Laudeman CP, Malkar NB, Krishnan R, Polowy K 2017 Structure-activity relationships of a series of echinocandins and the discovery of CD101, a highly stable and soluble echinocandin with distinctive pharmacokinetic properties. Antimicrob Agents Chemother 61:e01541-16. doi:10.1128/AAC.01541-16. 27919891 PMC5278707

[22] Krishnan BR, James KD, Polowy K, Bryant BJ, Vaidya A, Smith S, Laudeman CP 2017 CD101, a novel echinocandin with exceptional stability properties and enhanced aqueous solubility. J Antibiot (Tokyo) 70:130–135. doi:10.1038/ja.2016.89. 27507631

[23] Lepak AJ, Zhao M, VanScoy B, Ambrose PG, Andes DR 2018 Pharmacodynamics of a long-acting echinocandin, CD101, in a neutropenic invasive-candidiasis murine model using an extended-interval dosing design. Antimicrob Agents Chemother 62:e02154-17. doi:10.1128/AAC.02154-17. 29203480 PMC5786781

[24] Locke JB, Almaguer AL, Zuill DE, Bartizal K 2016 Characterization of *in vitro* resistance development to the novel echinocandin, CD101, in *Candida* species. Antimicrob Agents Chemother 60:6100–6107. doi:10.1128/AAC.00620-16. 27480852 PMC5038289

[25] Ong V, Hough G, Schlosser M, Bartizal K, Balkovec JM, James KD, Krishnan BR 2016 Preclinical evaluation of the stability, safety, and efficacy of CD101, a novel echinocandin. Antimicrob Agents Chemother 60:6872–6879. doi:10.1128/AAC.00701-16. 27620474 PMC5075098

[26] Sandison T, Ong V, Lee J, Thye D 2017 Safety and pharmacokinetics of CD101 IV, a novel echinocandin, in healthy adults. Antimicrob Agents Chemother 61:e01627-16. doi:10.1128/AAC.01627-16. 27919901 PMC5278714

[27] Zhao Y, Perez WB, Jimenez-Ortigosa C, Hough G, Locke JB, Ong V, Bartizal K, Perlin DS 2016 CD101: a novel long-acting echinocandin. Cell Microbiol 18:1308–1316. doi:10.1111/cmi.12640. 27354115 PMC5096055

[28] Arendrup MC, Meletiadis J, Zaragoza O, Jorgensen KM, Marcos-Zambrano LJ, Kanioura L, Cuenca-Estrella M, Mouton JW, Guinea J 2018 Multicentre determination of rezafungin (CD101) susceptibility of Candida species by the EUCAST method. Clin Microbiol Infect 24:1200–1204. doi:10.1016/j.cmi.2018.02.021. 29505881

[29] Chandra J, Ghannoum MA 2018 CD101, a novel echinocandin, possesses potent antibiofilm activity against early and mature *Candida albicans* biofilms. Antimicrob Agents Chemother 62:e01750-17. doi:10.1128/AAC.01750-17. 29133552 PMC5786756

[30] Hall D, Bonifas R, Stapert L, Thwaites M, Shinabarger DL, Pillar CM 2017 *In vitro* potency and fungicidal activity of CD101, a novel echinocandin, against recent clinical isolates of *Candida* spp. Diagn Microbiol Infect Dis 89:205–211. doi:10.1016/j.diagmicrobio.2017.07.007. 28826987

[31] Pfaller MA, Messer SA, Rhomberg PR, Jones RN, Castanheira M 2016 Activity of a long-acting echinocandin, CD101, determined using CLSI and EUCAST reference methods, against *Candida* and *Aspergillus* spp., including echinocandin- and azole-resistant isolates. J Antimicrob Chemother 71:2868–2873. doi:10.1093/jac/dkw214. 27287236 PMC5031917

[32] Pfaller MA, Messer SA, Rhomberg PR, Castanheira M 2017 Activity of a long-acting echinocandin (CD101) and seven comparator antifungal agents tested against a global collection of contemporary invasive fungal isolates in the SENTRY (2014) Antifungal Surveillance Program. Antimicrob Agents Chemother 61:e02045-16. doi:10.1128/AAC.02045-16. 28052853 PMC5328534

[33] Pfaller MA, Messer SA, Rhomberg PR, Castanheira M 2017 CD101, a long-acting echinocandin, and comparator antifungal agents tested against a global collection of invasive fungal isolates in the SENTRY 2015 Antifungal Surveillance Program. Int J Antimicrob Agents 50:352–358. doi:10.1016/j.ijantimicag.2017.03.028. 28689871

[34] Castanheira M, Messer SA, Rhomberg PR, Dietrich RR, Pfaller MA 2018 Activity of a long-acting echinocandin rezafungin (CD101) and comparator antifungal agents tested against contemporary invasive fungal isolates: SENTRY 2016, poster Friday-485 ASM Microbe, Atlanta, GA American Society for Microbiology, Washington, DC.

[35] Pfaller MA, Messer SA, Rhomberg PR, Schaefer BA, Castanheira M 2018 Activity of a long-acting echinocandin rezafungin and comparator antifungal agents tested against contemporary invasive fungal isolates: SENTRY 2018, poster 2400. IDWeek, San Francisco, CA. 10.1128/AAC.00099-20 PMC7179261 32015043

[36] Pfaller MA, Carvalhaes CG, Messer SA, Rhomberg PR, Castanheira M 2019 Activity of a long-acting echinocandin, rezafungin, tested against invasive fungal isolates collected worldwide, Poster 2115. IDWeek, Washington, DC.

[37] Clinical and Laboratory Standards Institute. 2017 M27 Reference method for broth dilution antifungal susceptibility testing of yeasts. Clinical and Laboratory Standards Institute, Wayne, PA.

[38] Clinical and Laboratory Standards Institute. 2018 M38 Reference method for broth dilution antifungal susceptibility testing of filamentous fungi, 3rd ed Clinical and Laboratory Standards Institute, Wayne, PA.

[39] Clinical and Laboratory Standards Institute. 2016 M57 Principles and procedures for the development of epidemiological cutoff values for antifungal susceptibility testing, 1st ed Clinical and Laboratory Standards Institute, Wayne, PA.

[40] Clinical and Laboratory Standards Institute. 2018 M60 Performance standards for antifungal susceptibility of testing yeasts. Clinical and Laboratory Standards Institute, Wayne, PA.

[41] Clinical and Laboratory Standards Institute. 2018 M59 Epidemiological cutoff values for antifungal susceptibility testing, 2nd ed Clinical and Laboratory Standards Institute, Wayne, PA.

[42] Pfaller MA, Diekema DJ 2012 Progress in antifungal susceptibility testing of *Candida* spp. by use of Clinical and Laboratory Standards Institute broth microdilution methods, 2010 to 2012. J Clin Microbiol 50:2846–2856. doi:10.1128/JCM.00937-12. 22740712 PMC3421803

[43] Pfaller MA, Messer SA, Woosley LN, Jones RN, Castanheira M 2013 Echinocandin and triazole antifungal susceptibility profiles of opportunistic yeast and mould clinical isolates (2010–2011); application of new CLSI clinical breakpoints and epidemiological cutoff values to characterize geographic and temporal trends of antifungal resistance. J Clin Microbiol 51:2571–2581. doi:10.1128/JCM.00308-13. 23720791 PMC3719648

[44] Arendrup MC, Jorgensen KM, Hare RK, Cuenca-Estrella M, Zaragoza O 2019 EUCAST reference testing of rezafungin susceptibility and impact of choice of plastic plates. Antimicrob Agents Chemother 63:e00659-19. doi:10.1128/AAC.00659-19. 31285230 PMC6709479

[45] Oxman DA, Chow JK, Frendl G, Hadley S, Hershkovitz S, Ireland P, McDermott LA, Tsai K, Marty FM, Kontoyiannis DP, Golan Y 2010 Candidaemia associated with decreased *in vitro* fluconazole susceptibility: is *Candida* speciation predictive of the susceptibility pattern? J Antimicrob Chemother 65:1460–1465. doi:10.1093/jac/dkq136. 20430790

[46] Castanheira M, Woosley LN, Diekema DJ, Jones RN, Pfaller MA 2013 Candida guilliermondii and other species of Candida misidentified as Candida famata: assessment by Vitek2, ITS sequence analysis and matrix-assisted laser desorption ionization–time of flight mass spectrometry in two global surveillance programs. J Clin Microbiol 51:117–124. doi:10.1128/JCM.01686-12. 23100350 PMC3536252

[47] Pfaller MA, Woosley LN, Messer SA, Jones RN, Castanheira M 2012 Significance of molecular identification and antifungal susceptibility of clinically significant yeasts and moulds in a global antifungal surveillance program. Mycopathologia 174:259–271. doi:10.1007/s11046-012-9551-x. 22580756

[48] Castanheira M, Messer SA, Jones RN, Farrell DJ, Pfaller MA 2014 Activity of echinocandins and triazoles against a contemporary (2012) worldwide collection of yeast and moulds collected from invasive infections. Int J Antimicrob Agents 44:320–326. doi:10.1016/j.ijantimicag.2014.06.007. 25129315

[49] Pfaller MA 2015 Invasive fungal infections and approaches to their diagnosis. Methods Microbiol 72:219–287.

[50] Pfaller MA, Rhomberg PR, Wiederhold NP, Gibas C, Sanders C, Fan H, Mele J, Kovanda LL, Castanheira M 2018 *In vitro* activity of isavuconazole versus opportunistic fungal pathogens from two mycology reference laboratories. Antimicrob Agents Chemother 62:e01230-18. doi:10.1128/AAC.01230-18. 30061288 PMC6153788

